# Public awareness of care pathways and available skill mix in NHS dental teams: a qualitative study

**DOI:** 10.1038/s41415-025-8491-z

**Published:** 2025-07-11

**Authors:** Abubakar Sha´aban, Francesca Mazzaschi, Andrew Dickenson, Anthony Cope, Elizabeth Doe, Warren Tolley, Adrian Edwards, Natalie Joseph-Williams

**Affiliations:** 41415402122001 Health and Care Research Wales Evidence Centre, Division of Population Medicine, Cardiff University, Cardiff, UK; 41415402122002 Directorate of Primary Care, Mental Health and Early Years, Welsh Government, Cardiff, UK; 41415402122003 Health and Care Research Wales Evidence Centre Public Partnership Group, Division of Population Medicine, Cardiff University, Cardiff, UK

## Abstract

**Introduction** Limited public understanding of the dental team's roles and confusion about accessing urgent dental care (UDC) and emergency dental services (EDSs) are barriers to efficient service use. Understanding and addressing these gaps is essential to improving patient access, enhancing experience and successfully integrating skill mix into dentistry.

**Aims** This study explores public awareness of the dental team in Wales and the processes for accessing UDC. It also seeks to identify participants' information needs and suggestions for improving awareness and understanding of the general dental team and UDC.

**Method** A qualitative study involving semi-structured interviews and focus groups was undertaken. Data were analysed thematically, capturing key themes in participants' responses.

**Results** We found limited public understanding of the different dental team member roles and participants lacked clarity on how and when to access UDC. Participants demonstrated a limited understanding of dental roles beyond the dentist, frequently using terms like ‘dental assistant' to describe various team members. Participants called for better education on the dental team and clearer guidance on what constitutes a dental emergency or how to access UDC.

**Conclusion** The findings reveal critical knowledge gaps about dental team member roles and UDC processes, which could play a key role in patients making informed choices about appropriate care pathways and using the available skill mix in the dental team. Targeted public education campaigns are necessary to address these gaps, improve patient confidence in seeking care from other dental team members, and enhance service efficiency.

**Supplementary Information:**

Zusatzmaterial online: Zu diesem Beitrag sind unter 10.1038/s41415-025-8491-z für autorisierte Leser zusätzliche Dateien abrufbar.

## Key points

- Public understanding of dental team roles and urgent care pathways is limited in Wales.
- Education campaigns are essential to improve awareness of dental services and skill mix models.
- Better understanding of roles could enhance acceptance of skill mix and patient confidence in diverse dental care.
- Addressing awareness gaps can improve service efficiency, access and job satisfaction among dental professionals.

**Supplementary Information:**

Zusatzmaterial online: Zu diesem Beitrag sind unter 10.1038/s41415-025-8491-z für autorisierte Leser zusätzliche Dateien abrufbar.

## Introduction

Demand for dental services far exceeds supply in the United Kingdom (UK) and a diverse staff with different skills and expertise (Table 1) has been considered one way to enhance overall capacity. Further, around 380,000 patients inappropriately attend UK general medical services with dental problems annually,^1^ placing additional strain on already pressurised care pathways. The changing mix of clinical dental staff, with a notable increase in dental therapists, highlights the evolving structure of the workforce. For example, in the five years^2^ to November 2024,^3^ the number of General Dental Council (GDC)-registered dental therapists in the UK doubled numerically and in proportion to the number of registered dentists, now represent roughly one for every six registered dentists. However, if individuals are expected to effectively navigate the NHS (National Health Service) dental system, knowing when, where and who to see for different dental concerns, and to be responsive to increased skill mix in general dentistry, they need a greater awareness and understanding of service structure and the specific responsibilities of each team member.^4^

**Table 1:** Agreed NHS Wales role descriptions

**Role**	**Description**
Dentist	University degree Registered with the GDC Seen as the team leader; dentists ‘own' the NHS dental contract Responsible for assessing mouth conditions, not just teeth but also the skin inside the mouth and throat Provide complex dentistry, such as tooth extraction, root canal, bridges, implants and dentures Some provide mouth surgery, such as removal of wisdom teeth
Dental nurse	Registered with the GDC Have a specific qualification from a tertiary college Works closely with the dentist, responding quickly to requests and keeps the surgery ready for use Responsible for decontamination of instruments and maintaining dental operating equipment Ensures all relevant materials and supplies are in place, including ordering stock Looks after patient records, including making notes when the dentist is examining a patient Can provide oral health advice to patients Some have extended roles (based on extra qualifications) to apply fluoride varnish
Dental hygienist	Registered with GDC Have a university qualification (e.g., a Bachelor's degree or diploma) Mainly responsible for scaling and polishing teeth and helping patients with their gum health Can give local anaesthetics for pain relief Can provide fluoride varnish and fissure sealants as part of the prevention of dental disease Take dental x-rays Take impressions of teeth
Dental therapist	Registered with GDC Have a university degree Work independently and can see patients without a dentist doing the initial assessment Offer some of the services like a hygienist e.g., scaling and polishing, applying materials to teeth, such as fluoride and fissure sealants Take dental x-rays Take impressions of teeth Undertaking fillings on baby and permanent teeth Putting in crowns Extract baby teeth
Dental technician	Registered with the GDC Hold a diploma from a tertiary college or university degree Dental technicians work directly with patients in a clinic alongside a dentist Make all appliances e.g., crowns, bridges, dentures, or children's orthodontic braces
Orthodontist	A qualified dentist who has undertaken additional specialist training in straightening teeth Registered with the GDC Usually only work with braces and most don't offer routine dentistry
Note: Role standards change over time; hence, it is essential to consult the GDC website and *Scope of practice* for the most up-to-date role descriptions.	

Recent reform plans for dental services in Wales^5^ and other UK nations^6^^,^^7^ promote a skill mix approach, encouraging patients to seek care from different members of the dental team according to their specific needs. This allows teams to release dentist time for new patient access or those with more complex needs. This strategy aims to enhance service efficiency, reduce pressure on dentists and ensure patients receive timely care. It may also support overall recruitment and retention initiatives in dentistry. Significant amendments were also introduced to the NHS dental contract in 2022, removing administrative barriers preventing some dental care professionals, such as dental therapists, from operating to their full scope of practice.^8^ Subsequent guidance^9^ provides information on how to achieve this, including dental therapists and dental hygienists providing care where it is within their scope of practice.

However, a key barrier to successfully implementing this approach is limited public awareness of the various roles within the dental team and the range of services they offer.^10^ Public engagement with dental services often focuses on the dentist as the sole care provider, leading to underutilisation of other skilled dental professionals, such as dental hygienists, therapists and dental nurses, who play significant roles in preventive care, periodontal treatment and routine restorative procedures.^9^ Previous studies from English NHS dental patients^11^ and internationally^12^ show that public knowledge of the dental team and their roles is limited, often unaware of the full range of services provided by dental therapists or the contributions made by dental hygienists to oral health maintenance. We also know that many patients lack clarity on how and where to seek urgent dental service, meaning they access inappropriate care pathways, such as general medical practice.^13^^,^^14^^,^^15^

These knowledge gaps present a considerable challenge to achieving a more integrated and efficient dental service that provides the right care for the patient in the right location in a timely way. With dental reform underway in Wales, we sought to explore public awareness of care pathways and skill mix in the Welsh context to help identify information gaps and needs that need to be addressed. It is part of a broader qualitative study that examines priorities for dental care services in Wales^16^ to ensure reform plans align with public needs.

## Aims

The study aimed to explore public awareness of general and urgent NHS dental services in Wales. Specific objectives were to explore: a) what members of the public know about NHS general dental services and the dental team; b) their understanding on how and when to access urgent dental care; and c) information gaps and needs.

## Methods

### Design

Qualitative research design employing interviews and focus groups.

### Sample

Participants included individuals eligible to access NHS General Dental Services in Wales who were 18 years or older at the time of recruitment. The number of participants was determined by data saturation,^17^ samples used in other healthcare service delivery qualitative studies,^18^ and purposive sampling.^19^ Data saturation means data were collected until no new significant themes arose from interviews. A purposive sampling approach^20^ was used to ensure a diverse and representative sample based on demographic factors, including sex, age, health board area, education, employment, disability status, income, ethnicity and NHS dental registration status (meaning a person is recognised by the dental practice as one of their NHS patients and will see them when required, including routine examinations and any necessary treatment). We also asked participants three questions exploring components of digital literacy.^20^

### Recruitment

Participants were recruited through social media platforms, mainly via the ‘Xʹ accounts of Health and Care Research Wales, Health and Care Research Wales Evidence Centre and PRIME Centre Wales. Interview and focus group opportunities were advertised separately. Posters summarising the study were included in the social media posts alongside a link to the study's webpage, where potential participants could access detailed participant information sheets for both opportunities. Interested individuals completed an online screening and demographic survey to register their interest and allow us to sample purposively. The purposively selected sample of participants were contacted and asked to provide a convenient time for the interview or were provided login details for the focus group. Consent forms were then sent to participants for review, signature and return before the interview or focus group.

### Data collection

Data (video and audio) were collected through virtual semi-structured interviews and a focus group conducted via Microsoft Teams or Zoom between November 2023 and January 2024 (interviews) and April 2024 (focus group). Participants were given an information sheet outlining key dental service definitions before the interviews to aid discussion. An interview schedule was developed with stakeholder input and guided the discussions. Key topics included public understanding of the ‘dental team', the roles involved in providing needs-based dental care, and expectations for access to general and emergency dental services.

Before data collection, we sought clarification from AD and WT on the different roles (including dentist, dental nurse, dental hygienist, dental therapist, dental technician and orthodontist) as a guide to assessing participants' awareness and understanding (see Table 1 for agreed role descriptions used in NHS Wales).

### Data analysis

The audio portion of the data were transcribed verbatim by a Cardiff University-authorised transcription service. Transcripts were analysed using thematic analysis^21^(AS/FM) facilitated by NVivo 12 Software.^22^ Interview and focus group data were analysed together. The analysis involved coding the data, developing a thematic framework, charting data into the framework and interpreting the key themes. Initial coding frameworks were discussed and refined through discussion among researchers (AS/FM/NJW) before being applied to the entire dataset.

### Public involvement

Our public partner co-author (AC) was involved in the development of the interview schedule to ensure the questions were accessible and the topics relevant to the public were addressed. Their input was instrumental in shaping the research focus and ensuring that the study addressed issues that mattered most to participants.

### Ethical approval

Cardiff University School of Medicine Research Ethics Committee (SMREC 23.71) granted favourable ethical approval for this study.

## Results

### Participants

Overall, 44 participants were recruited: 35 for interviews and nine for the focus group. Of these, 39% (n = 17) identified as female, 57% (n = 25) as male and 4% (n = 2) as non-binary. Participants' age groups ranged from 18-24 (n = 4), 25-34 (n = 18), 35-44 (n = 4), 45-54 (n = 7), 55-64 (n = 4), 65-74 (n = 5), to 75-84 (n = 2), with the largest proportion in the 25-34 age group. Participants came from all seven health boards in Wales and represented diverse backgrounds regarding disability status, educational attainment, employment status and personal income (see Supplementary Table 1). A total of 77% (n = 34) were currently registered with an NHS dentist, 16% (n = 7) were not, and 7% (n = 3) were unsure. Ethnic backgrounds included white (n = 26), Black, African, Caribbean or Black British (n = 12), multiple ethnic group (n = 4), Asian or Asian British (n = 1), and one participant who preferred not to disclose their ethnicity.

Self-reported digital literacy varied, including participants' abilities to use applications, set up video calls and resolve basic technical issues. A total of 38 participants (86%) agreed or strongly agreed that they could independently use applications or programs (such as Zoom) on devices like mobile phones, computers, or tablets without needing assistance. Regarding video calls, 37 participants (84%) agreed or strongly agreed that they could set up video calls on their electronic devices without seeking help. Similarly, 37 participants (84%) reported confidence in their ability to solve or figure out basic technical issues independently.

### Key findings

Three key themes (and eight sub-themes) emerged during data analysis. Exemplary quotes are included below; further quotes are included in Supplementary Table 2.

#### Awareness and understanding of the general dental service team

Participants reflected on their awareness of the different members of the dental team, their understanding of their roles and how this compares to their understanding of other healthcare teams and professionals.

##### Limited awareness of dental team roles

Most participants demonstrated a limited understanding of the broader dental team and were unaware of all key roles (see Table 1). The most mentioned roles were the dentist, dental nurse and dental hygienist. However, around one-third of participants reported limited understanding of the team beyond the role of ‘dentist':

- ‘*I heard about the dentist. It's all about the dentists to me'* (P33)
- ‘*So, I feel apart from a dentist, the dental specialist. I don't know. It is your dentist, and the dental specialist should be able to look after my dental care'* (P35).

Only a few participants mentioned roles such as ‘dental technicians' (one participant) and ‘orthodontists' (two participants). Despite being a key role highlighted in NHS skill mix guidance,^9^ no participants identified or expressed awareness of the ‘dental therapist' role. Some participants referenced ‘dental surgeons' and ‘specialists,' acknowledging that these professionals typically provide care in specialised settings and often require referrals.

Although not formally recognised as part of the general dental service team, many participants perceived administrative staff as integral to the ‘dental team.' These staff members play a crucial role in coordinating appointments, assisting patients in navigating their care, and facilitating communication between patients and dental professionals:

- ‘*So this and also the receptionist…I would regard them as, you know, a core member of the team. Because they are the ones responsible for receiving patients and then communication with the dentist'* (P134).

One participant felt that patients were also a core part of the ‘dental team':

- ‘*I mean, those who are involved, like the patients, the community support groups...so, to me, when we talk about teams, it shall be not just the service providers, but the service users as well*' (P75).

##### Limited understanding of dental team roles

Most participants seemed aware that the different team members performed different roles and described tasks that would be done by a dental hygienist (e.g., scale and polish), a therapist (e.g., x-rays or teeth impressions) or a dental technician (e.g., making bridges or dentures). However, they were uncertain who performed them and what their role was called:

- ‘*Okay, I think the dental team consists of a couple of, number of, people ranging from the dentist and, and the [dental] hygienist and we do not know how to name them, but I know that they have different roles. Yeah, they do not do the same thing'* (P37)
- ‘*My understanding is the dental assistant can either be the dental technician or the dental nurse. I think it depends on the practice*' (P34).

Many participants used the term ‘dental assistant' to refer to anyone present during their appointment other than the dentist, regardless of their specific qualifications or job title. This label was often applied broadly, encompassing various roles, indicating a lack of distinction between different qualified roles within the dental team:

- ‘*So thinking about the dental team, I would usually refer to the dentist, who is really a core part of the dental team. And also there is the dental assistants that help the dentist when he is about to perform a procedure'* (P4).

One participant did not feel that the team members had different titles or provided different services, instead seeing them all as ‘dental service providers':

- ‘*They're still dentists, they're still part of the dental services. So although you've listed them out separately, for me, and for most laypeople, they see them as one family…they don't see them as different…they might be different individuals, but they're not different service care providers*' (P75).

##### Less understanding of the ‘dental team' compared to other healthcare teams

Some participants reported that they had much less understanding of the dental team roles and how they work together as a team when compared to other healthcare teams that they interact with:

- *‘I would say I have a much greater understanding of other teams than a dental team. I didn't realise there was so much available to a dental team because I've never been offered it. And I've never experienced it. And I've never noticed these other positions exist'* (P73)
- ‘*I definitely have less understanding of what a dental team comprises of and their roles, compared to like you say, a GP [general medical practitioner] practice. I'd have far more understanding if I was to go to a GP practice and they said “you're best off speaking to the physiotherapist who comes here once a week rather than the GP”. Whereas, a kind of similar example for the dentist I wouldn't know why or who I'd be seeing and why I wouldn't be seeing the dentist'* (P174).

##### Importance of understanding in supporting skill mix

When discussing their lack of awareness and understanding of the different dental team members, many participants expressed a need for better information about the different roles and expertise available in the general dental setting:

- ‘*It's kind of all tied together really, you know, making people really aware of what these different roles do and their expertise…they might have been through the same kind of training, and letting people know that'* (P174).

Most of these participants reflected that if they had this information and a better understanding of the different roles, they would be more receptive to seeing different members of the dental team:

- ‘*Sometimes, at the doctors, it's very appropriate to see a nurse, a physiotherapist. You don't always have to see the GP…if we were properly educated as to who can do what, and then we think “oh, well, that's okay, I'm seeing the dental nurse. I'm not actually seeing the dentist today, but dental nurses know about and do this”'* (P74).

#### Understanding of urgent and emergency dental services

Participants reflected on their understanding of urgent dental care (UDC) and emergency dental services (EDSs). They described what their actions would be in urgent or emergency dental situations, including who they would contact, where they would seek care and how they would determine the appropriateness of UDC or EDSs for the issue they were experiencing.

##### Deciding when something is a dental emergency

Despite many participants not knowing who to contact or where to go in a dental emergency, many participants reflected on various factors that would help them identify if their situation was a dental emergency.

These factors included constant bleeding, severe pain that cannot be managed by regular painkillers, post-oral surgery complications, unexpected injury (e.g., life-threatening accidents, domestic violence), when there might be an infection and a risk of sepsis, and if they feel that they needed support or treatment within less than 24 hours.

However, some participants reported feeling unsure about what an emergency is:

- ‘*And also, I don't really know what, what constitutes an emergency'* (P74)
- ‘*Not really, but I know that in, in an emergency dental care, you have to, you know, book an appointment, probably sometimes a few days before'* (P37).

##### Uncertainty on how to access emergency dental services

Many participants reported uncertainty about who to contact or where to go if they had an urgent or emergency dental situation:

- *‘Oh, now you're going into a very complicated area…I mean, that's a whole can of worms…emergency dental services is still not clearly defined or understood by the patients and members of the public, and I suspect even the dental professions themselves between one dental profession and another dental profession*' (P75).

Several participants reported that they would contact their dentist in the first instance. This was often because of trust and their established relationship with the dentist. This was also seen as a sign-posting opportunity to access the correct EDS service:

- ‘*My first port of call is always going to be to my dentist because I have much more trust in my dentist. And then I will get referred from my dentist to someone else'* (P75).

Only a few participants said that they would use the NHS 111 service (telephone triage and advice) to access EDSs; this was usually a back-up option when the dental practice was closed:

- ‘*If it is out of hours, if it is on the weekend, or when my dental practice is closed, then I will go into 111 services to get access to emergency dental care'* (P75).

Some participants said that they would attend A&E (accident and emergency) with a dental emergency:

- ‘*Well, for me, I think our first point of call would be A&E at the hospital, and from there, they would…I think from there they would direct us to the actual section that we are supposed to be at'* (P52).

#### Information needs

When describing their awareness and understanding of general services and EDSs, participants also identified key information gaps and needs and explained how this information could be delivered.

##### What do the public need more information on?

There was a strong need for improved communication and information. This includes: a) better information about different general dental service team members and their roles; b) better information about what constitutes a dental emergency versus urgent or routine dental care; c) better information about who to contact and where to go in a dental emergency situation.

##### How should this information be delivered?

Participants made various suggestions for how this information could be delivered to the public and the format of this information. This included: a) government/NHS campaigns; b) community outreach; c) school and youth programmes; d) traditional advertising methods; and e) personal involvement in raising dental care awareness. Throughout, participants were mindful of ensuring all materials were accessible and inclusive and of the personal role they could play in actively engaging in promoting dental care awareness e.g., within their families or communities.

## Discussion

Our findings show limited public awareness of the different dental team roles and participants lacked clarity on how and when to access urgent care. While most participants had heard of the dentist, dental nurse and dental hygienist, very few were aware of dental technicians and no participants were aware of dental therapists. Significantly, while participants could describe different dental tasks conducted by different team members and recall some of the team members, they remained unclear on who undertook these, often categorising various dental professionals under the broad label of ‘dental assistants'. We also found that many patients were uncertain about when and how to access EDSs; although, some felt they could judge when that care might be appropriate. Our findings indicate significant knowledge gaps regarding the full range of dental services and roles, limiting patients' abilities to make fully informed choices about which care pathway they should choose and which professional they should see.

### The role of greater awareness and understanding in successfully deploying skill mix

Our findings align with previous work, which also highlighted gaps in public awareness of dental professionals' roles. Marshall^23^ found that the public often had a narrow view of the dental team, typically associating dental care with dentists alone, and found that the public was generally unfamiliar with the dental therapist and dental hygienist roles. Combined with our findings, this points to a broader issue of visibility and communication between the dental profession and the public. The British Society of Dental Hygienists and Therapists recognises the need for greater awareness of their roles, as evidenced by their webpage offering downloadable leaflets and posters (for adults and children) to display in practices and clinics.^24^

**Fig. 1:**
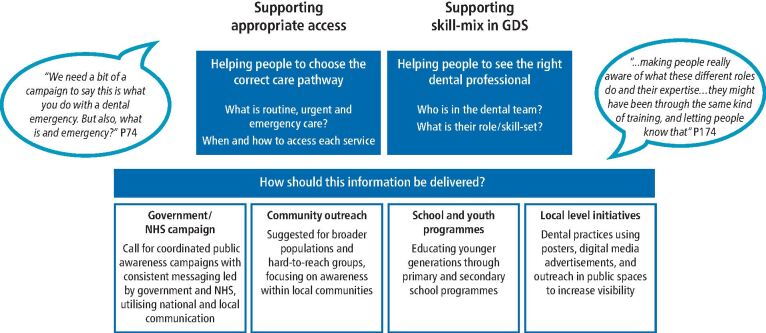
Public information needs for appropriate care access and use of skill mix

A study on patients' experiences of skill mix changes in primary medical care suggests that clear and effective communication about the roles is crucial to enhancing patient trust in additional healthcare practitioners (such as nurse practitioners, pharmacists, or physiotherapists).^25^ Successful skill mix in dentistry will rely partly on patients' acceptance of seeing other dental professionals in place of their usual appointments with a dentist. Many patients are unaware that other qualified professionals could provide specific treatments, which can affect trust and acceptance of care from non-dentist providers. Improving understanding of the dental team is essential for informed decision-making about which dental professional to seek care from and enhancing patient confidence in their care. Lack of awareness or misunderstanding of roles may delay people seeking appropriate care, contribute to unnecessary reliance on dentists, and create inefficiencies in accessing routine and specialised services. To encourage public acceptance of skill mix and promote booking appointments with alternative dental team members to increase capacity and enhance access, the first crucial step is to improve public understanding of: a) the identities of dental team members; and b) the scope of their roles (see Fig. 1).

### Supporting patients to access appropriate care pathways

The public remains uncertain about accessing UDC and EDSs. While some participants mentioned contacting their dentist as a first option, others were unaware of alternative services, with only a few recognising NHS 111 as an option. Some were also unsure what constituted a dental emergency and when to seek urgent care. There is a clear need for better public education on accessing EDSs and recognising what a dental emergency is.

The confusion around accessing urgent dental services identified in our study is a recurrent theme in UK research. For example, an urgent dental care evidence review by Public Health England^26^ found widespread uncertainty about where to seek help for dental emergencies. Many participants defaulted to contacting their regular dentist or visiting A&E, reflecting confusion about accessing appropriate EDSs. This is consistent with our findings, in which participants expressed similar uncertainty about navigating EDS options. Similarly, the recent publication by Innes-Taylor *et al.*^27^ highlights gaps in the provision of unscheduled dental care experienced by individuals with UDC needs and offers steps to improve the UDC system in England.

### Addressing information gaps to support skill mix and informed choice about appropriate care

Participants expressed a strong need for better information about dental team roles, what constitutes urgent and emergency dental care, and accessing these care pathways (see Fig. 1). This shows that current communication strategies regarding dental services are not meeting patient needs. Without clear, accessible information, patients may feel uncertain about who is treating them, when they need care and where to go. Improving the information provided to patients will empower them to make informed decisions about who they see and where they seek care, reduce confusion and enhance their overall experience with dental care. It could also reduce unnecessary reliance on medical services or emergency services when routine dental care would suffice.

Information about skill mix and care pathways should be targeted at multiple levels. Governments should lead public awareness campaigns to educate the public on the diverse roles within dental teams, clarifying how this approach can enhance access and efficiency without compromising quality. Outreach approaches should be used to reach those who are less heard and seen in NHS dentistry. School- or youth-based programmes could educate patients early in their life course, supporting them in making informed dental care choices as they transition to adult services. The dental profession can reinforce these efforts by actively educating patients about different team roles and ensuring consistent communication of their qualifications. Dental clinics should also highlight how skill mix roles can address specific care needs efficiently. Importantly, a coordinated communication campaign with unified messaging via these different routes could build public trust and understanding of these evolving roles in dental care.

### Strengths and limitations

This study has explored the views of diverse participants from multiple health boards across Wales, age groups, ethnic backgrounds, employment/incomes and disability status. While the diversity of the sample supports the relevance of these findings to other dental care settings, further research is needed to confirm transferability across different contexts. Although the sample size of 44 participants may appear modest, it is appropriate for this qualitative study, where we achieved data saturation and focused on capturing the depth and richness of individual experiences rather than broad generalisation. The qualitative methods employed in this study provided valuable insights into participants' needs and the underlying reasons, enabling exploration of potential solutions. Although the participant group was relatively diverse, it is important to acknowledge a potential bias arising from the self-selecting nature of the sample, particularly due to the digital data collection methods. Future research should aim to include individuals who are less likely to self-select for participation and use in-person data collection approaches to ensure broader representation.

## Conclusion

This study highlights significant gaps in public awareness of dental team roles and access to UDC and EDSs. Participants demonstrated limited awareness beyond the dentist's role, often conflating various dental team members or expressing uncertainty about their specific functions. These findings highlight the need for targeted public education campaigns that: a) raise awareness of the dental team's structure and roles; and b) the appropriate routes for accessing urgent care. Addressing these knowledge gaps is key to achieving skill mix and supporting appropriate access to urgent dental services. Through better communication, we can enhance patient confidence in receiving care from a broader range of dental professionals and improve the efficiency of general and EDSs. Such developments could also contribute to increased cost-effectiveness of dental services and enhanced job satisfaction among dental care professionals, fostering a more sustainable and motivated workforce.

## Supplementary Information

Supplementary Tables 1-2 (PDF 313KB)

## Data Availability

De-identified data supporting this study's findings may be made available upon reasonable request to the corresponding author, subject to approval by the relevant ethics committee and in compliance with institutional and data protection regulations.
