# Supplementary material for: Public awareness of care pathways and available skill mix in NHS dental teams: a qualitative study
Source: Br Dent J. 2025 Jul 11;239(1):39–45. doi: 10.1038/s41415-025-8491-z (PMC12254029; doi:10.1038/s41415-025-8491-z)
Supplement: Supplementary file 1 — Supplementary Tables 1-2 (PDF 313KB) [file 41415_2025_8491_MOESM1_ESM.pdf]

Supplementary Table 1 Demographic characteristics of participants (n=44)

Research ID	Gender M = Male F - Female	Age Range (years)	Current NHS dental registration status	Local health board area	Disability Status	Highest educational level attained	Employment status	Personal income last year (£)	Ethnicity
**Interview participants (n=35)**									
P1	M	25 – 34	Not sure	Cardiff and Vale University Health Board	Chronic illness	University level qualification	Self-employed	1 to 9, 999	Asian or Asian British
P3	F	65 – 74	Currently registered with an NHS dentist	Hywel Dda University Health Board	None	University level qualification	Retired	10, 000 to 24, 999	White
P4	F	45 – 54	Currently registered with an NHS dentist	Aneurin Bevan University Health Board	Neurodivergent (e.g., autism, ADHD)	University level qualification	Employed part-time	25, 000 to 49, 999	White
P5	F	25 – 34	Currently registered with an NHS dentist	Aneurin Bevan University Health Board	None	University level qualification	Student (full-time)	10, 000 to 24, 999	White
P6	F	45 – 54	Not currently registered with an NHS dentist	Cardiff and Vale University Health Board	Chronic illness	University level qualification	Employed full-time	75, 000 to 99, 999	White
P7	M	25 – 34	Currently registered with an NHS dentist	Cardiff and Vale University Health Board	None	University level qualification	Employed full-time	25, 000 to 49, 999	White
P8	F	65 – 74	Currently registered with an NHS dentist	Betsi Cadwaladr University Health Board	None	University level qualification	Self-employed	10, 000 to 24, 999	White
P9	F	55 – 64	Currently registered with an NHS dentist	Betsi Cadwaladr University Health Board	None	University level qualification	Retired	Prefer not to say	White
P12	M	25 – 34	Currently registered with an NHS dentist	Cwm Taf Morgannwg University Health Board	None	University level qualification	Employed full-time	10, 000 to 24, 999	Black, African, Caribbean or Black British

P13	M	35 – 44	Not currently registered with an NHS dentist	Cardiff and Vale University Health Board	Mobility impairment	University level qualification	Self-employed	10, 000 to 24, 999	Mixed or Multiple ethnic groups
P18	M	25 – 34	Currently registered with an NHS dentist	Swansea Bay University Health Board	None	University level qualification	Employed part-time	10, 000 to 24, 999	White
P22	M	75 – 84	Currently registered with an NHS dentist	Betsi Cadwaladr University Health Board	Hearing impairment	School or college leaver qualification	Retired	10, 000 to 24, 999	White
P23	M	25 – 34	Currently registered with an NHS dentist	Cwm Taf Morgannwg University Health Board	Chronic illness	University level qualification	Employed part-time	25, 000 to 49, 999	Black, African, Caribbean or Black British
P24	M	55 – 64	Not sure	Betsi Cadwaladr University Health Board	Stroke survivor - some anxiety, mental health issues and concentration difficulties	University level qualification	Retired early due to stroke, unable to work	25, 000 to 49, 999	White
P27	M	25 – 34	Currently registered with an NHS dentist	Swansea Bay University Health Board	None	University level qualification	Employed full-time	50, 000 to 74, 999	Black, African, Caribbean or Black British
P28	M	25 – 34	Currently registered with an NHS dentist	Swansea Bay University Health Board	None	School or college leaver qualification	Employed part-time	25, 000 to 49, 999	Black, African, Caribbean or Black British
P30	M	18 – 24	Currently registered with an NHS dentist	Swansea Bay University Health Board	None	University level qualification	Student (full-time)	25, 000 to 49, 999	White
P33	M	18 – 24	Currently registered with an NHS dentist	Aneurin Bevan University Health Board	None	School or college leaver qualification	Employed part-time	10, 000 to 24, 999	Black, African, Caribbean or Black British
P35	M	25 – 34	Currently registered with an NHS dentist	Betsi Cadwaladr University Health Board	None	School or college leaver qualification	Employed full-time	75, 000 to 99, 999	White

P36	M	18 – 24	Currently registered with an NHS dentist	Swansea Bay University Health Board	None	School or college leaver qualification	Employed part-time	25, 000 to 49, 999	Black, African, Caribbean or Black British
P37	F	25 – 34	Currently registered with an NHS dentist	Not Applicable	None	University level qualification	Employed part-time	25, 000 to 49, 999	Black, African, Caribbean or Black British
P38	F	18 – 24	Currently registered with an NHS dentist	Powys Teaching Health Board	Visual impairment	School or college leaver qualification	Self-employed	25, 000 to 49, 999	Black, African, Caribbean or Black British
P43	F	45 – 54	Currently registered with an NHS dentist	Not sure	Mobility impairment	No formal qualifications	Unable to work due to illness or disability	10, 000 to 24, 999	White
P44	M	25 – 34	Not currently registered with an NHS dentist	Powys Teaching Health Board	Mental health condition	University level qualification	Employed part-time	50, 000 to 74, 999	Black, African, Caribbean or Black British
P45	F	35 – 44	Not sure	Cardiff and Vale University Health Board	Neurodivergent (e.g., autism, ADHD)	School or college leaver qualification	Unable to work due to illness or disability	1 to 9, 999	White
P49	F	25 – 34	Currently registered with an NHS dentist	Cardiff and Vale University Health Board	None	School or college leaver qualification	Employed part-time	50, 000 to 74, 999	Black, African, Caribbean or Black British
P52	M	25 – 34	Currently registered with an NHS dentist	Cardiff and Vale University Health Board	None	University level qualification	Employed full-time	50, 000 to 74, 999	Mixed or Multiple ethnic groups
P53	F	25 – 34	Currently registered with an NHS dentist	Cardiff and Vale University Health Board	None	University level qualification	Employed part-time	50, 000 to 74, 999	Black, African, Caribbean or Black British
P55	F	45 – 54	Currently registered with an NHS dentist	Aneurin Bevan University Health Board	None	School or college leaver qualification	Retired	100, 000 or more	White
P71	M	45 – 54	Currently registered with an NHS dentist	Hywel Dda University Health Board	None	University level qualification	Employed full-time	Prefer not to say	White
P72	M	75 – 84	Not currently registered with an NHS dentist	Aneurin Bevan University Health Board	Type 1 Diabetes	School or college leaver qualification	Retired	£10, 000 to £24, 999	White

P73	F	45 – 54	Currently registered with an NHS dentist	Powys Teaching Health Board	Hearing impairment	University level qualification	Employed full-time	£25, 000 to £49, 999	White
P74	F	55 – 64	Currently registered with an NHS dentist	Cardiff and Vale University Health Board	None	University level qualification	Employed part-time	£1 to £9, 999	White
P75	M	45 – 54	Currently registered with an NHS dentist	Cardiff and Vale University Health Board	None	School or college leaver qualification	Prefer not to say	Prefer not to say	Mixed ethnicity
P76	F	35 – 44	Not currently registered with an NHS dentist	Swansea Bay University Health Board	None	University level qualification	Employed full-time	£25, 000 to £49, 999	White
**Focus group participants (n=9)**									
P2	M	55 – 64	Currently registered with an NHS dentist	Aneurin Bevan University Health Board	None	University level qualification	Retired	£10, 000 to £24, 999	White
P21	M	65 – 74	Currently registered with an NHS dentist	Cardiff and Vale University Health Board	None	University level qualification	Prefer not to say	£10, 000 to £24, 999	White
P25	M	65 – 74	Currently registered with an NHS dentist	Cardiff and Vale University Health Board	None	University level qualification	Retired	£25, 000 to £49, 999	White
P88	M	25 – 34	Currently registered with an NHS dentist	Aneurin Bevan University Health Board	None	University level qualification	Employed full-time	£25, 000 to £49, 999	Black, African, Caribbean or Black British (African background)
P122	M	25 – 34	Currently registered with an NHS dentist	Cardiff and Vale University Health Board	None	University level qualification	Employed full-time	£50, 000 to £74, 999	White
P134	non-binary	25 – 34	Currently registered with an NHS dentist	Swansea Bay University Health Board	None	University level qualification	Self-employed	£10, 000 to £24, 999	Mixed or Multiple ethnic groups (White and Black Caribbean)

P104	non-binary	25 – 34	Currently registered with an NHS dentist	Swansea Bay University Health Board	None	University level qualification	Self-employed	£25, 000 to £49, 999	Prefer not to say
P174	M	35 – 44	Not currently registered with an NHS dentist	Swansea Bay University Health Board	None	University level qualification	Employed full-time	£50, 000 to £74, 999	White
P173	F	65 – 74	Not currently registered with an NHS dentist	Swansea Bay University Health Board	None	School or college leaver qualification	Employed part-time	£10, 000 to £24, 999	White

Supplementary Table 2 Qualitative themes, sub-themes, and exemplar quotes

Theme	Sub theme	Category (if relevant)	Example Quotes[1]
Awareness and understanding of the general dental service team	***Limited awareness of the dental team***		"I could only identify three roles. One being the receptionist, the other being the dentists themselves, and then the third one being the oral hygienist or something like that. I think those are the only three roles that I could actually identify in there." P8 Interview
			"The dentist, the hygienist, the dental nurse, the receptionist, the, and then whatever specialty you get referred to as well." P45 Interview
			"*I'd include the receptionist, as part of the team, because there's a lot they can reassure people on and spot when someone comes in. Obviously, the main provider is the dentist himself or herself, but [they] are an important part of that as well.*" P24 Interview
	Limited understanding of dental team roles		*"Okay. As far as I'm aware, there's the, the big man at the top [dentist's name] who owns the practice. He's got a team of dentists who work underneath him. They've got a couple of hygienists, and they've got assistants."* P22 Interview
			"Obviously you've got the dentist. I was thinking dental hygienist as well and I don't know whether they're the same as the dental assistant that [Workshop participant] mentioned." P6 Interview
	Limited understanding of the 'dental team' compared to other healthcare teams		*"If I visited my GP surgery, and I saw, for example, a nurse practitioner…or a phlebotomist…then I would absolutely know that if they had concerns or needed to check something that they were going to be going off to the doctor, having their meetings, deciding what the best way forward is. But because I guess they're working together, they're in the same building, they might even just call their colleague in. Whereas in the dental world, I suppose I don't have so much of that picture." P21 Focus group*
	Importance of understanding in supporting skill-mix		"there has to be that comfort and trust. You know having trust in the expertise of your own dentist, that's, that's, that's a thing." P27 Interview

[1] In the exemplar quotes, 'P' denotes participant, and 'I' denotes interviewer.

Understanding of urgent and emergency dental services	***Uncertainty on how to access EDS***		*"That is part of what I was describing, there are no more information as to how people would go about that. Because yeah, I do not know how to access* it." P12 Interview
			"Contact your, your dentist that you registered with." P76 Focus group
			"So, the reason why I say we need to contact our dentist is because our option may not be perfect. So, I think the dentist is the person that is in a perfect place to give you legit guidance on what you're supposed to do at that time." P22 Interview
			"there's the [Hospital in Wales]. I know that there's a dental department there, you know, where people can go to for emergency treatment. I always thought it was for people without a dentist…." P45 Interview
	Deciding if something is a dental emergency		"Well an abscess, for example, where potentially it's serious it's infected and there's a risk of sepsis." P24 Interview
			"the dental pain is such that it's really distressing and needs urgent treatment that might not be available in a, a normal high street dental provider." P24 Interview
**Information Needs**	***What do the public need more information on?***	***Better information about the different team members and their roles***	"If we were properly educated as to who can do what... I'm seeing the dental nurse. I'm not actually seeing the dentist today, but dental nurses know about and do this." (P74 Focus group)
			"… it's kind of all tied together really, you know, making people really aware of what these different roles do and their expertise…they might have been through the same kind of training, and letting people know that." P174 Focus group
		Better education about EDC, what constitutes a dental emergency and where to go	"We need a bit of a campaign to say this is what you do with a dental emergency. But also, what is an emergency? So, if I've got toothache, that can probably wait until tomorrow. Or I might take some paracetamol, and it might go away." P74 Interview
			"We should create awareness whereby people are learning about the emergency dental services... how to handle dental emergencies." (P35 Interview)

		**Greater awareness of services and clarity of access was also important to participants**.	*"…I also feel that people should be educated, like we should create awareness whereby people are learning about the emergency dental services. That is going to help people seek immediate care. And [know] how to handle dental emergencies."* P35 Interview
			"…lack of awareness, because a lot of individuals might not be aware of their existence, or anything that has to do with emergency, so them lacking knowledge can also make them to be ignorant about what is happening to themselves, or maybe people around them. So, we should try to create more awareness and teach people on emergency dental services." P27 Interview
	What should this information look like and how should it be delivered?	***Community Outreach***	*"In some local communities or in some places, it's very hard for them to access this care. And I know there are people, there are some people who are not even aware of this care. So I think my suggestion is that awareness should be created for people who are maybe in the rural areas, to know about this care."* P72 Interview
		School and Youth Outreach for Dental Awareness	"There is a place of community outreach, you know, you have to conduct outreach in schools." P27 Interview
			"All right, I think, number one, awareness should be created but in high school seminars." P38 Interview
		Inclusive education options	*"You educate people in the best way for them as individuals, you know, because we're not all the same. Some people can't read, [some] are blind and deaf, how do you do it then. You have to look at it in a holistic way."* P72 Focus group
		Government / NHS Campaign	*"…obviously then there's the broader picture if you like, where you utilise the Welsh [Government's] communications processes..." P72 Focus group*
		Improving awareness and communication	*"So one of my bugbears, there's no communication in certain circumstances, [they] don't actually let you know what it is they want you to do." P72 Focus group*
			"I mentioned earlier on about proper communication with each patient, and I'm always talking about the patient because delivery should be very patient-centred." P28 Interview

		**Traditional advertising methods**	*"I guess they could like put up posters and things like that in surgeries and hospitals and just advertisement, really." P55 Interview*
			"And I think to achieve those opportunities by going round, I think we should go for, by creating awareness in worship centres, in market squares, I think in pharmacies, in hospitals, in communities, to let people there aware of those services, to be aware of what is actually going on." P23 Interview
		**Personal Involvement in Dental Care Awareness**	*"Okay, to me, I feel I can be involved in the shaping of the dental care services in the society by also teaching people around me. Also, teaching people around me the benefits of, of knowing anything, knowing things about emergency health, emergency dental care services [inaudible 00:42:48] like educating people more on the emergency." P35 Interview*
